# PReS-FINAL-2357: Effects of anti-melanocyte stimulating hormone in murine pristine-induced lupus

**DOI:** 10.1186/1546-0096-11-S2-P347

**Published:** 2013-12-05

**Authors:** T Peixoto, S Mello, D Botte, S Carrasco, M Vendramini, C Goldenstein-Schainberg

**Affiliations:** 1 Reumatologia, Faculdade de Medicina, Universidade de Sao Paulo, Sao Paulo, Brazil

## Introduction

Alfa-melanocyte stimulating hormone (α-MSH) has a variety of biological functions such as downregulation of pro-inflammatory pathways, reduction of skin delayed-type hypersensitivity and blockage of leukocyte migration. Inhibition of experimental disease models development including inflammatory bowel disease and rheumatoid arthritis has been shown, however the immunomodulatory and anti-inflammatory effects of α-MSH on murine lupus remain undetermined.

## Objectives

To evaluate the effect of α-MSH analogue (NDP α-MSH) on pristane-induced murine lupus.

## Methods

Thirty-five BALB/c mice were injected with 0.5 ml intraperitoneal (IP) pristane for lupus-like model induction and 5 age/gender matched control mice were given saline. Pristane-induced lupus animals received daily IP saline (n = 5) or treatments with 3.1 mg/kg/d chloroquine (n = 10), 1.25 mg/kg/d NDP α-MSH (n = 10) or 2.5 mg/kg/d NDP α-MSH (n = 10). Prior and 180 days after induction, clinical and laboratorial lupus-like parameters were examined. Sera ANA was tested by IF using Hep2 cells. Statistical analysis was performed by Mann-Whitney and Fisher test and P < 0,05 considered significant.

## Results

Arthritis in both hind legs and large amounts of lipogranulomas in peritoneal cavity were observed in all lupus-like animals in contrast to all controls. By visual observation, all lupus animals treated with both doses of α-MSH had significant less amount and lower size lipogranulomas. Mean arthritis score in 5 untreated mice, 9 animals treated with chloroquine and 8 with α-MSH 2.5 mg/kg/d was 5.2, 3.33 and 3.1 respectively. Remarkably, mean arthritis score of animals treated with α-MSH 1.25 mg/kg/d was 1.6, significantly lower than untreated mice (1.6 vs 5.2, p = 0.0291). ANAs were negative in sera from all 40 animals before pristane lupus injection; 180 days after induction, ANAs remained negative in normal mice but became positive in all 5 (100%) untreated lupus animals, 7 (77%), 4 (50%) and 3 (35%) lupus models treated with chloroquine, α-MSH 2.5 mg/kg/d and α-MSH 1.25 mg/kg/d (100% vs 35%, p = 0,0256), respectively. Before the end of the experiment, by day 150, 3 animals died: 1 treated with chloroquine and 2 with higher doses of α-MSH.

## Conclusion

NDP α-MSH promoted improvement of clinical and serological parameters in pristane-induced murine lupus suggesting a potential role for this drug in human SLE.

## Disclosure of interest

None declared.

